# Is Balance Training Using Biofeedback Effective in the Prophylaxis of Falls in Women over the Age of 65?

**DOI:** 10.3390/brainsci13040629

**Published:** 2023-04-06

**Authors:** Teresa Sadura-Sieklucka, Leszek Tomasz Czerwosz, Ewa Kądalska, Marcin Kożuchowski, Krystyna Księżopolska-Orłowska, Tomasz Targowski

**Affiliations:** 1 National Institute of Geriatrics, Rheumatology and Rehabilitation, 02-637 Warsaw, Poland; 2 Department of Respiration Physiology, Mossakowski Medical Research Institute, Polish Academy of Sciences, 02-106 Warsaw, Poland

**Keywords:** balance training, posturography, force plate, visual motor feedback, biofeedback, virtual reality, centre of foot pressure, COP, inertia forces, physiotherapy

## Abstract

The paper aims to investigate the usefulness of training in improving mobility and reducing the risk of falls of patients with osteoarthritis by using a force plate and virtual reality as rehabilitation tools. The study involved 72 women randomly divided into two equal groups: the force plate training group, which underwent virtual balance training with visual motor feedback, and the gym training group, which received conventional balance training only. The functional balance assessment was performed before and after the rehabilitation by computerized posturography in a relaxed upright position with open and closed eyes, with visual motor feedback. In the FPT group in the feedback measurement, the mean radius of sways was 30% smaller after rehabilitation (*p* < 0.00002); the feedback coordination coefficient was more than 10% bigger after rehabilitation (*p* < 0.001) and reached 92%, which is excellent for elderly people. Total stagnation and stumbling reported by patients decreased after rehabilitation compared to the first examination. Both tested forms of training can contribute to reducing the risk of falls. However, a more significant improvement was obtained in the force plate training group perhaps because the physical effort on a force plate trains the precise movements needed to reposition the centre of gravity without generating excessive inertia forces responsible for loss of balance and falls. Perhaps the most desirable method of intervention is to train a person’s ability to perform slow but definite body movements.

## 1. Introduction

The growing number of elderly people is associated with the occurrence of numerous diseases related to the physiological ageing process; osteoarthritis is one of them [1]. The global impact of osteoarthritis is being observed, it will increase with increasing incidence of obesity and increasing life expectancy. Osteoarthritis constitutes a significant social burden, associated with polypharmacy and increased mortality [2,3]. Pain, stiffness of joints, muscle weakness, instability or uncertainty while walking, and physical inactivity are problems frequently reported by patients [4,5,6]. All of these symptoms increase the risk of falls. Their very frequent consequences include trauma, hospitalization or institutionalization, which entails significant social costs [7,8]. There is an urgent need to develop effective strategies to reduce the risk of falls and identify people at risk [9,10]. The recommended physiotherapy used in the prevention of falls includes strength exercises, focused mainly on improving the strength of the lower limbs, and classic balance exercises, which should take into account the gradual increase in difficulty: reducing the support plane, reducing the stability of the base, limiting eye control, etc., [11,12,13,14]. Exercises on a force plate is an interesting idea for teaching the patients to control physical effort in order to precisely change the position of the centre of gravity without generating excessive inertia forces, which are often the direct cause of a fall, as they cause the point of application of the pressure (COP) to be shifted beyond the foot area. The visual-motor feedback (biofeedback) in posturography consists in linking the image observed by the person standing on a force plate with his/her motor activity. Over the years virtual reality devices have become increasingly popular and affordable. This makes them a good way to continue self improvement by the patient at home, as well as to monitor their progress [15]. This makes a force plate and virtual reality a rehabilitation tool that can be used in balance training [16]. Training with the use of VR technology can become more effective in improving the mental state, mobility and overall quality of life in older people [17].

The aim of this study is to investigate the usefulness of training on a force plate in improving the mobility and reducing the risk of falls of patients with osteoarthritis in the lower limbs.

## 2. Materials and Methods

The inclusion criteria were as follows: age over 65 years, knee osteoarthritis, lack of balance disorders in clinical examination. We excluded patients with sight and hearing disorders limiting the possibility of testing and training, neurological diseases, cognitive problems, positive Romberg test result, severe pain in the knee joints that prevents training. Seventy-two women, divided randomly into two groups, were included in the study. The force plate training group (FPT), which performed virtual balance training on force plate (Pro-Med), consisted of 36 patients with average age 63 ± 8; and the gym training group (GT) consisted of 36 patients with average age 65 ± 8.

The study used a questionnaire containing personal data as well as questions regarding the number of stumbles and falls that occurred in the previous two months. Pain was assessed using the Laitinen scale, in which the patient rated four determinants: pain intensity, frequency of pain occurrence, use of analgesics and limitation of motor activity. The subjective assessment of problems in the knee joints was made using the Lequesne pain-functional index.

Functional performance and risk of falling were determined using the Timed Up and Go (TUG) test. The Four Square Step Test (FSST) was used to assess the dynamic balance, i.e., to maintain the correct stable posture while moving.

The static balance test was performed using a force plate in the relaxed position with the eyes open (EO) and the eyes closed (EC), and with visual motor feedback [16]. Measurements were taken in the previous 32 s.

The essence of the posturographic measurement is the determination of a trajectory of the centre of foot pressure (COP) locations and the analysis of its shape. From each statokinesiogram, the following variables were calculated:

R: average radius of all COP points from the centre of the coordinate system,

L: COP trajectory length.

For feedback measurements a K% index was calculated: the feedback coordination coefficient K% was equal to the proportion of the number of samples located inside the figure to the total number of measurement samples while the figure was visible to the patient. The K% index determines the effectiveness of the patient’s efforts to keep the marker within the virtual geometric figure.

The feedback measurements and the training sessions involved the observation by a patient of a graphic marker (a small square) on a 55-inch monitor screen. The marker position on the screen reflected the instantaneous position of the COP point on the horizontal plane on the force plate. Simple geometrical figures were displayed for several seconds successively on the screen. Visual feedback was used for the training and rehabilitation of the balance system [18,19,20].

The screen was placed in front of the eyes of a patient within the range of good visibility. Objects visible on the screen were clear and large, there was no problem of poor visibility; if necessary, the patient put on his own distance glasses.

The scale of displaying figures and displacements of the marker on the patient’s screen was similar to the real displacements of the COP point on the force plate base. The angular size of the section of any length on the force plate, seen by a person with a height of 150–180 cm, was approximately the same as on the screen as seen from a distance of 200 cm. Therefore, it can be considered that the size of the virtual image was “natural”. Pinsault and Vuillerme described the virtual image scale effect in feedback during a quiet state on a force plate [21]. The visual conditions during training were the same as during feedback diagnostic measurements in an upright position.

**Figure 1:**
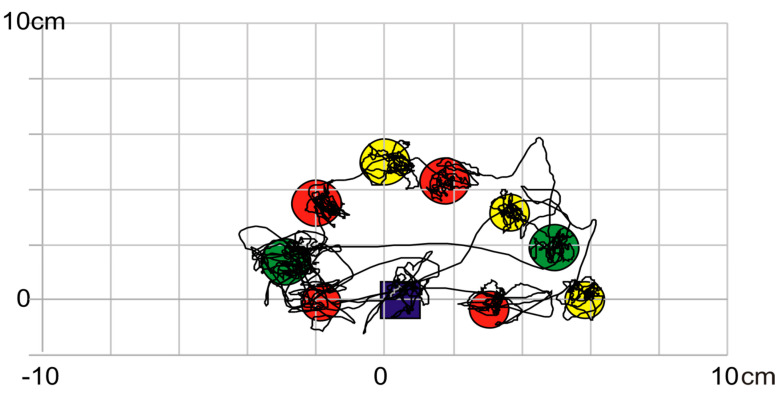
A statokinesiogram: the trajectory of the COP movements on the ground XY plane together with the positions of virtual objects on the screen. Please note that whereas the individual objects appear on the screen sequentially, the above picture shows them all together.

In the feedback diagnostic measurements, the position of the COP was the result from the random body tilts and the intentional body movements. These movements were supposed to correct the position of the body and the COP position so that the marker indicating the position of the COP point, remained within the centrally located virtual target. In the force plate training, visual targets in the form of geometric figures were placed in different places on the screen and distant from the central position by a few centimeters (Figure 1).

All participants underwent a comprehensive rehabilitation, which lasted for 3 weeks. In addition, patients in the gym training (GT) group performed 30 min of conventional training, including stretching, strength and balance exercises. The women randomized to the force plate training (FPT) group practiced stability training on the force plate as described above. Exercises were performed every day, 5 days a week, for 3 weeks. In the following weeks, the difficulty of force plate training was increased by moving the virtual figures away from the central position and changing the order of virtual objects. Single training consisted of three sessions with a total duration of 6 min. The training sessions were repeated with increasing difficulty and adjusted to the physical abilities of the patient.

Each person who took part in the full cycle of tests was assessed three times: (I) before starting the training, (II) after comprehensive rehabilitation, extended by a series of 15 training sessions on a force plate or in the gym and (III) three months after the end of the exercises.

The statistical analysis concerned the parameters of statokinesiograms performed with the eyes open and closed and with feedback. The intragroup analysis was performed using the parametric t-test for paired data and in the intergroup analysis the t-test for unpaired (independent) data was used.

Some of the patients stopped training and we did not take further measurements, which resulted in their complete removal in the paired statistics (pairwise). In unpaired comparisons, all patients participating in the given stage were taken into account.

A comparison was made between examinations I and II and then between examinations II and III. The first comparison was used to assess the impact of rehabilitation on the measured variables and the second was related to the durability of the results of the achieved fitness.

## 3. Results

In the FPT group (Table 1), the number of incidents of stumbling reported by patients in examination III decreased significantly compared to examination I. No statistically significant differences were found in the GT group for the number of instances of stumbling. The Lequesne functional index was significantly lower in examination II compared to examination I in the FPT group. No statistically significant differences were also found in the GT group. In the FPT group, the Laitinen scale (*p* < 0.004) behaved very similarly to the Lequesne index (*p* < 0.02), the impact of rehabilitation (I vs. II) was much stronger and it was also marked in the GT group.

In the Timed Up and Go test (TUG), no differences were found between examinations I and II in the FPT and GT groups. In the Four Square Step Test (FSST) scale, there was a small decrease in the test execution time in examination II compared to examination I in both groups.

The mean values of the posturographic variables with the standard deviation are presented in Table 2. These variables are calculated from the statokinesiograms for each type of measurement in each examination separately in both groups. The intragroup differences between examinations I, II and III, as well as intergroup differences between the FPT and GT groups, are shown.

The mean radius of sways R (Table 2) and the length of the COP trajectory L in measurements taken with the eyes open (EO) and closed (EC) during examinations I, II and III did not differ. There were also no intergroup differences between the FPT and GT groups.

In the FPT group, the feedback radius in examination II differed greatly from the radius in examination I (2.92 ± 0.66 and 4.57 ± 2.01, respectively; *p* < 0.00002). The difference was expressed in the shortening of the mean radius of sways. This means that the body’s stabilization is more effective and the range of movements of the virtual marker inside the virtual square is reduced. In the GT group, this change was not observed.

In posturographic examination II, a large difference in the radius of sways between the FPT and GT groups (2.92 ± 0.66 and 4.44 ± 2.35 respectively, *p* < 0.0006) was observed. In the FPT group, the positive effect of training was maintained—examinations II and III showed no difference. Patients from the GT group showed no difference between examinations I vs. II and II vs. III. Only the radius R changed in the feedback, while the length L did not.

The K% coefficient in the FPT group in examination II was significantly higher than in examination I (92.1% ± 10.0% and 80.0% ± 18.2%, respectively; *p* < 0.001). This corresponds to the results with the sway radius. The K% coefficient in the GT group did not change when comparing examinations I vs. II.

The analysis of the mean values of the sway radius R and the K% coefficient in the feedback measurement was completed with the analysis of individual values of R and K% combined with the χ^2^ (chi-square) statistical test. In the vast majority of patients, the K% coefficient increased and the R radius decreased.

**Figure 2:**
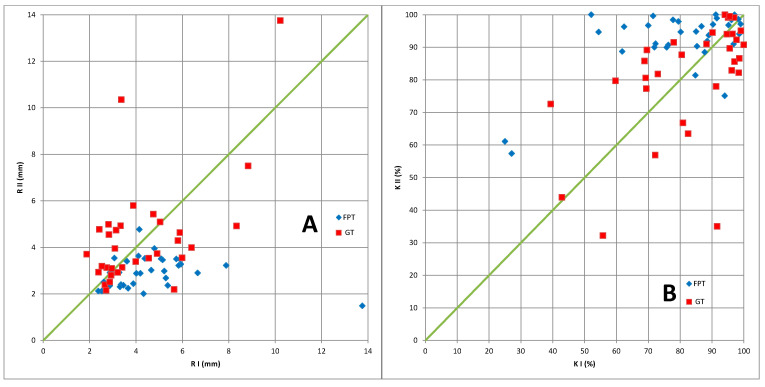
The scatter plot for individual patients: (**A**) radius values of sways in examination I versus II; (**B**) K% coefficient in the measurement with the feedback in examination I versus II. The horizontal axis (RI or KI) is related to examination I; and the vertical axis (RII or KII), to examination II. The areas of the plots are split with diagonal lines. In panel A, below the bisector, there are points for which RII < RI, i.e., for which the sway radius in examination II decreased compared to examination I; above the bisector, there are points with RII > RI. In panel B, above the bisector, there are points for which KII > KI, i.e., for which K% in examination II increased compared to examination I; below the bisector, there are points with KII < KI, i.e., K% coefficient in examination II was smaller than in examination I. Each point represents a patient: the blue diamonds are the FPT group, the red squares are the GT group.

**Figure 3:**
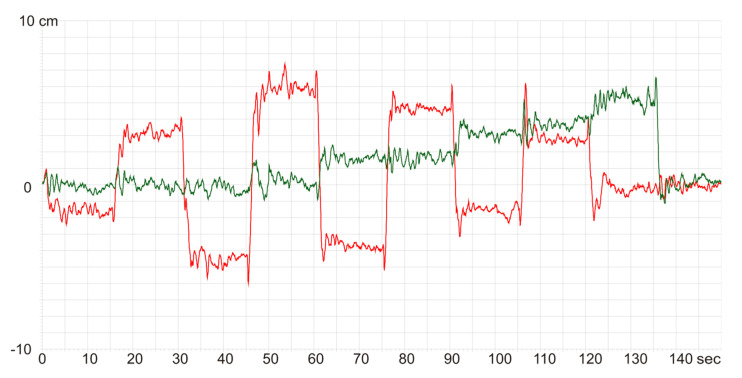
The x(y) and y(t) stabilograms for the force plate training. The green stabilogram shows movements in the *y*-axis in the sagittal—anteroposterior (AP) plane; the red one, in the *x*-axis, i.e., in the frontal—mediolateral plane (ML).

Panel A in Figure 2 shows a scatter plot of the sway radius R values for individual patients in the feedback measurement in examinations I and II in both groups: FPT and GT. In panel B there is a scatter plot of the K% coefficient. Figure 3 shows the trajectory of the COP point movement in the form of a stabilogram, i.e., graphs of the x(t) and y(t) coordinates of the COP point.

There are significantly more patients in the FPT group whose R decreased in examination II compared to I (proportion 30:4—see Table 3). These are the points marked as blue diamonds below the diagonal line (panel A). There was perfect symmetry in the GT group (16:16). The χ^2^ statistic was 11.4, *p* < 0.0007. Similarly, there are significantly more patients in the FPT group whose R increased in examination II compared to examination I (29:5). These are the points marked as blue diamonds above the diagonal line (panel B). There was perfect symmetry in the GT group (16:16). The χ^2^ statistic was 9.5, *p* < 0.002.

The χ^2^ analysis is consistent with the previous comparative analysis of the mean values of the radius R and the K% coefficient (see Table 2).

## 4. Discussion

Fall prevention requires the implementation of appropriate prevention strategies that should take into account the patient’s health and functional problems, as well as external co-morbidities. A significant proportion of the patients assessed by us, in the absence of clinical features of balance disorders and an increased risk of falls, reported tripping and loss of balance, and a few reported falls, suggesting that reaching the age of 65 alone contributes to the fall problem. For all elderly people, aerobic, stretching, coordination and balance exercises, as well as strength exercises, are recommended [11]. According to a meta-analysis by Sherrington et al. [22] in fall prevention, we can see better relative effects in programs that improve balance, are more intense and do not include a walking program. There is increasing use of virtual reality in rehabilitation [23,24,25,26]. The question to ask is how the patient should perform the exercises in order for them to be effective.

It has been found in this study that training with the use a force plate and exercises with visual feedback are effective tools that can be used to improve the ability and speed of multidirectional movements, resulting in a reduction in patient-reported loss of balance and improved performance in most of the tests used. Omni-directional tilts of body on a force plate, which are a form of exercise mainly for the lower limbs, teach the trainee to successfully target the COP marker at the virtual target—a square or circle. This effectiveness is related not only to the ability to move the centre of gravity and keep the body leaning for a few seconds by straining the core muscles but also to the ability to avoid rapid alternating movements of the COP by quietly and slowly activating or releasing the muscle forces. It should be remembered that the uncontrolled activation or release of the muscle force causes the generation of rapidly changing inertia forces, which cause rapid additional movement of the virtual marker on the screen, representing the movements of the COP point on the real plane of support. These additional displacements occur in the opposite direction to the actual movement of the body’s centre of gravity—in accelerated motion and in the direction of movement—in the braking motion [27]. In the feedback measurements, the virtual marker representing the COP can show the patient a false position of his/her body, because the COP position does not indicate the centre of gravity, but the point of application of the resultant pressure force composed of the gravity and inertia forces. As a result of the incorrect interpretation of the COP marker positioning by the patient, the results of the feedback measurement become worse than the results from the physical skills and fitness of the examined person.

Training on a force plate undoubtedly develops the ability to shift the body without generating excessive inertia forces. This is shown by the results achieved by people exercising on a force plate in examinations II and even III compared to I.

A postural measurement with feedback is taken to enable a person standing on a force plate and watching the computer screen to notice a shift in his/her COP before there is increased tension in the postural muscles or the eyesight notices the shift of the head. The computer measurement system is more sensitive to small COP shifts than human senses; therefore, one should expect better body coordination under feedback conditions than with open eyes. Studies of Komar and Czerwosz [27] and Imaizumi et al. [28] explain the large ambiguity of the impact of feedback measurements in limiting the range of sways.

In Figure 3, the x coordinate corresponds to the deflections in the frontal plane and the y coordinate represents moving in the sagittal plane. The graphs of x(t) and y(t) are slightly jagged, due to the instability of the work of the core muscles. Sharp “teeth” result from the influence of inertial forces disturbing the measurement of the actual body deflection that is changing slowly. Inertia forces are responsible for the rapid changes in the position of the COP point. They change rapidly producing sharp spikes in alternating directions. Changes in the value and direction of inertia forces result from muscle tremors that occur with the increased effort of the core muscles. In the course of training, the patient learns not only to use the strength of the leg muscles adequate to the need for deflection but also to master the heterogeneity of muscle strength.

According to the experimental studies, visual feedback can influence postural control in healthy adults, both young and old, as well as in patients with balance disorders [29,30]. It should be noted again that our research material included elderly women with partial motor disabilities due to osteoarthritis and there were essentially no persons with imbalances other than the normal impairment of age-related balance control. Consequently, we did not expect a significant improvement in balance in our patients. It was indeed so, comparing the postural variables in examination I vs. II with the eyes open and closed, no statistically significant differences were obtained.

In both study groups, after three weeks of comprehensive rehabilitation, a slight reduction in the test execution time is visible, but only in the FSST test; and in both study groups, this change is statistically significant. Similar results, but on the basis of other diagnostic tests, were reported by Maranesi et al. [17]. The change in direction of movement that occurs in this test is very often the cause of loss of balance and in the worst case the cause of a fall. In the second fall risk assessment (TUG) test, no such difference was observed. We pay attention to the specificity of the TUG, which is not related to the ability to perform movements in changing directions. Dite W et al. found that FSST is a sensitive and specific test for the able-bodied population as well as for those at risk of falling [31]. Our patients are people without any imbalances. The FSST test result in our work indicates an improvement in the ability to quickly change direction while lifting the foot, which translates into a reduction in the risk of falling in patients exercising with a force plate. The high sensitivity and specificity of this test was also found in the work of Whitney et al., in the group of patients with a vestibular disorder [32]. Exercises consisting in tilting the body in various directions, conducted for a period of three months, successfully adapted the neuromuscular system of people exercising on a force plate with feedback to minimize the emerging horizontally directed forces of inertia at the time of acceleration and braking. In this way, these people reduced the horizontal component of the forces acting on the body in their movements. This ability reduces the possibility of skidding; however, the friction force on unstable and slippery surfaces may be too low to resist the horizontal forces in walking. People trained to perform body movements without generating large inertia forces have a chance to reduce the tendency to roll over. The exercises described here were of a stationary nature, they consisted only of intense deflections of the centre of gravity. This knowledge can further improve the effectiveness of exercise, also as part of e-Health training, which may be more cost-effective for some groups of older people [33].

Limitations in the study are related to the small population of subjects. Further research requires expanding the research group and including men in it.

## 5. Conclusions

In this sample, it was found that there was an improvement in both forms of training, which can contribute to reduce the susceptibility for falls. However, greater improvement in the conducted examinations was obtained in the FPT group perhaps because the physical effort on a force plate trained the precise movements needed to reposition the centre of gravity without generating excessive inertia forces responsible for loss of balance and falls. Perhaps the most desirable method of intervention is to train a person to be able to perform slow but definite body movements; this requires further research.

## Figures and Tables

**Table 1:** Clinical data in two groups: FPT and GT.

	FPT					GT				
Examination (N)	I (28)	II (28)	III (27)	Comparison of Examinations		I (29)	II (29)	III (28)	Comparison of Examinations	
				I vs. II	II vs. III				I vs. II	II vs. III
Lequesne index	12.6 ± 3.9	10.0 ± 4.2	9.5 ± 3.9	*p* < 0.02	ns	11.6 ± 5.7	9.1 ± 6.1	9.7 ± 6.9	ns	ns
Laitinen scale	6.3 ± 1.9	4.4 ± 2.3	4.0 ± 2.1	*p* < 0.004	ns	6.1 ± 3.3	4.2 ± 3.2	4.8 ± 3.8	*p* < 0.04	ns
TUG	7.5 ± 1.6	6.9 ± 1.3	6.9 ± 1.0	ns	ns	7.6 ± 2.1	6.8 ± 1.1	6.8 ± 1.04	ns	ns
FSST	10.0 ± 2.0	9.1 ± 1.5	8.9 ± 1.5	*p* < 0.05	ns	10.5 ± 3.7	8.9 ± 1.9	8.8 ± 1.8	*p* < 0.05	ns
Number of stumbling	9.1 ± 11.0	---	2.8 ± 4.2	*p* < 0.02 *	---	6.9 ± 10.5	---	4.0 ± 6.4	ns *	---
Number of falls	0.2 ± 0.6	---	0.0 ± 0.2	ns *	---	0.3 ± 0.8	---	0.1 ± 0.3	ns *	---

FPT = force plate training group, GT = gym training group, TUG = Timed Up and Go test, FSST = four square step test scale. Comparisons of the results: I—before training, II—immediately after full cycle of training and III—three months after training. Examinations I versus II and then II versus III were compared with a t-test for paired data. * Examination I versus III was compared instead of comparison I versus II only for number of cases of stumbling and number of falls, as these variables were not measured in examination II. There was no comparison of examinations II vs. III for these variables.

**Table 2:** Mean values with standard deviations of posturographic variables calculated for each type of measurement in each examination separately in both groups.

FPT						GT					
Examination (N)	I (36)	II (34)	III (29)	Comparison of Examinations		I (36)	II (32)	III (28)	Comparison of Examinations		Group Comparison FPT vs. GT
Eyes open EO				I vs. II	II vs. III				I vs. II	II vs. III	
R_mm	3.82 ± 1.24	3.32 ± 0.86	3.77 ± 1.24	ns	ns	4.28 ± 2.01	3.93 ± 1.50	3.70 ± 0.98	ns	ns	I, II, III: ns
L_cm	34.4 ± 7.8	35.3 ± 7.5	34.3 ± 9.2	ns	ns	34.6 ± 9.0	36.3 ± 8.4	34.9 ± 9.3	ns	ns	I, II, III: ns
Eyes closed EC				I vs. II	II vs. III				I vs. II	II vs. III	
R_mm	4.80 ± 1.57	4.29 ± 1.40	4.56 ± 1.42	ns	ns	4.98 ± 2.06	4.81 ± 1.58	4.21 ± 1.02	ns	ns	I, II, III: ns
L_cm	45.9 ± 12.4	46.7 ± 13.0	44.9 ± 14.3	ns	ns	47.8 ± 15.5	53.6 ± 25.5	47.0 ± 14.4	ns	ns	I, II, III: ns
Feedback FB				I vs. II	II vs. III				I vs. II	II vs. III	
R_mm	4.57 ± 2.01	2.92 ± 0.66	3.12 ± 0.92	*p* < 0.00002	ns	4.14 ± 1.95	4.44 ± 2.35	3.56 ± 1.01	ns	ns	I, III: ns II *p* < 0.0006
L_cm	46.7 ± 17.9	44.4 ± 9.4	41.7 ± 10.1	ns	ns	44.6 ± 18.7	49.9 ± 25.6	42.6 ± 14.60	ns	ns	I, II, III: ns
K%	80.0 ± 18.2	92.1 ± 10.0	93.4 ± 7.5	*p* < 0.001		84.1 ± 16.3	81.2 ± 17.8	88.8 ± 9.3	ns	ns	I, III: ns II *p* < 0.003

FPT = force plate training group, GT = gym training group, R—average radius, L—COP trajectory length, K% = coefficient for feedback measurement. The intragroup differences between examinations I and II, and II and III were statistically tested—the results are in columns named “Comparison of examination I vs. II and II vs. III, separately for FPT and GT groups. The result of comparison between FPT and GT groups is given in column “Group comparison FPT-GT”. The intergroup measurements are compared for examinations I, II and III separately.

**Table 3:** Variables: mean sway radius R and K% coefficient for feedback measurement for all individuals in examinations I and II. The cells contain the number of patients meeting the given inequality.

	R			K%		
	R_II_ < R_I_	R_II_ > R_I_	Total	K_II_ < K_I_	K_II_ > K_I_	Total
FPT	30	4	34	5	29	34
GT	16	16	32	16	16	32
Total	46	20	66	46	20	66
	χ^2^ = 11.4 *p* < 0.0007			χ^2^ = 9.5 *p* < 0.002		

FPT = force plate training group, GT = gym training group, R = mean sway radius for feedback measurement, K% = coefficient for feedback measurement.

## Data Availability

The data presented in this study are available on request from the corresponding authors.
